# Forensic Entomologists: An Evaluation of their Status

**DOI:** 10.1673/031.013.7801

**Published:** 2013-08-10

**Authors:** Paola Magni, Silvia Guercini, Angela Leighton, Ian Dadour

**Affiliations:** 1 Centre for Forensic Science, University of Western Australia, Crawley, Western Australia; 2 Faculty of Political Sciences “Roberto Ruffilli”, University of Bologna, Forlì-based, via Giacomo della Torre 1, 47100Forlì, Italy

**Keywords:** accreditation, education, National Academy of Sciences report, questionnaire

## Abstract

The National Academy of Sciences (2009) published a review charting several key recommendations on strengthening the forensic sciences as an entity as part of an initiative put forth by the USA Congress to streamline and improve the quality of the forensic sciences and their impact on the judiciary process. Although the review was not totally inclusive, many of its sentiments have permeated into all the forensic sciences. The following paper is designed to determine who is practicing the science of forensic entomology, and in what capacity, by questioning practicing forensic entomologists about the type of education obtained, their countries' standards and accreditation processes, as well as general demographic information such as age and gender. A 28-question survey was sent out to 300 forensic entomologists worldwide in 2009. Of the 70 respondents, 80% had a formal education (either Masters or PhD), and 66% published their research. Approximately 50% of respondents were involved in the delivery of expert evidence and writing up case reports, and countries were actively involved with accrediting personnel, facilities, and entomology kits. Many discrepancies within the reported practices and accreditation processes highlight the need for the adoption of a standard code of practice among forensic entomologists.

## Introduction

Forensic entomology is a subset of the forensic sciences whereby information and samples of insects and associated arthropods are gathered and analysed to help draw conclusions on legal matters from a crime scene. The origins of this science have been dated as far back as the early 13^th^ century (Catts and Goff 1992; Amendt et al. 2004, Gennard 2007), but incorporation of forensic entomology as an important component to case work was not recognised until the start of the 20^th^ century (for a brief review, see Amendt et al. 2004). The modern practice of forensic entomology encompasses areas of urban entomology, stored product entomology, and medico-legal entomology (Catts and Goff 1992). To a lesser extent, forensic entomologists can also be involved in veterinary entomology, livestock entomology, entomotoxicology, and human and insect DNA (Byrd et al. 2010).

While there have been great strides over the last century as technology and science has evolved, there must be continued support and acceptance by both academics and practitioners as they work alongside the police and legal authorities to adapt and progress forensic entomology into the modern era. As insect specimens are being recognised as integral to the physical evidence at a crime scene, similar to fingerprints and DNA, more court systems globally are requiring the services of the forensic entomologist as an expert witness. In courts of the United States, including the Supreme Court, a methodology by which the witness is held to ‘expert’ standards is colloquially known as the ‘Daubert Criteria,’ and ensures that all experts, regardless of field of practice, are held to the standard that a witness qualified as an expert by knowledge, skill, experience, training, or education, may testify in the form of an opinion (or otherwise) will have sufficient data, be based on reliable principles and methods, and would have applied the principles via the scientific method (Daubert vs. Merrell Dow Pharms., Inc., 509 U.S. 579, 584–587). The Daubert Criteria have proved a worthy addition to the US judiciary system, giving all areas of scientific endeavor a rigorous foundation that provides more objective and reliable evidence interpretation in a legal setting (Tomberlin et al. 2011).

Additionally, a report was published by the National Academy of Sciences (2009) charting several key recommendations on strengthening the forensic sciences as an entity as part of an initiative put forth by the US Congress to streamline and improve the quality of the forensic sciences and their impact on the judiciary process. Interestingly, the report did not include many of the peripheral disciplines such as forensic entomology; nevertheless, the sentiments are congruent.

The National Academy of Sciences report indicated that there were vast inequalities that existed on every level of jurisdiction and agency, from local municipalities through to federal. These inequalities included funding, access to proper instrumentation, skilled personnel, certification, accreditation, and even oversight disparities. These differences make the efficacy of practice among current disciplines in forensic science difficult to say the least. The report stipulated, “It is clear, however, that any approach to overhauling the existing system needs to address and help minimize the community's current fragmentation and inconsistent practices”(page 6). The report also highlighted that many of the forensic science disciplines were not standardized; there was no congruency in the certification of practitioners, nor was there any standardized accreditation process for laboratories or other forensic facilities. Moreover, their findings pointed out that most jurisdictions did not require accreditation or certification of either the practitioner or their facilities, and if standards were in place, there was no meaningful enforcement. These inadequacies clearly show that the evidentiary reliability of the expert witness in all fields of forensic science is in serious danger.

As the work of a forensic entomologist may in fact be carried out by pathologists, or police officers as well as entomologists, Amendt, et al. (2007) outlined such a set of desirable practices based on the European Association for Forensic Entomology's 2005 protocol. This publication supplied a framework that encouraged a high level of competency and, presumably, genre continuity. However, outside of listing the desirable practices and methodology, and despite specifically admitting that a “broad range of professionals” (p. 100) may partake in the collection of evidence, the article stopped short in advising that the discipline needs to develop and implement globally accepted qualifications or accreditations. Indeed, comparative to the *how* of forensic entomology, there is a dearth of information about *who* practices forensic entomology.

In fact, the introduction to Byrd and Castner (2010) highlights this blight, stating that a survey of forensic entomologists worldwide would provide an interesting perspective, presumably one that gives an idea of who comprises the global community of forensic entomologists. The last known accumulation of information concerning forensic entomologists was compiled by Lord and Stevenson (1986), and as such, the information provided was limited by the technology of the time. Sixty-two persons spanning 6 countries responded to their request for inclusion into their database. Today, this database is further augmented via various associations and organisations updating their respective internet and social media sites; however, other than listing of name, education, and occasionally the organisation the member is working with, little information is catalogued.

Given the repeated calls for standardisation of practice in several recent publications (Leung 2006; Amendt et al. 2007; Tomberlin et al. 2011), the scarcity of information about who is practicing the science of forensic entomology and in what capacity, and the continually increasing savoir fare surrounding this discipline by all members of the judicial arena, the focus of this paper was to assimilate some of this information from a survey sent to more than 300 forensic entomologists worldwide in 2009. This survey was devised with assistance from specialists in forensic entomology, sociology, and criminology. It was created with the aim of specifically quantifying and understanding the type of education obtained by the practicing forensic entomologist, their countries standards and accreditation processes, as well as general demographic information such as age and gender. The aim of the paper was to gain insight into the nature of the field of forensic entomology and to utilise the information found as a mechanism to indicate the changes required to become a unified and accredited science.

## Materials and Methods

The 28-question form was accompanied by a letter of introduction outlining the purpose of the survey and implied consent as well as contact information (available from P. Magni upon request). The questionnaire was in English, and presented to the participants at the VII European Association for Forensic Entomology Meeting, as well as distributed via mail to all members of the European Association for Forensic Entomology, the North American Forensic Entomology Association, Gruppo Italiano per l'Entomologia Forense, and the Forensic Entomology Yahoo Group list (http://tech.groups.yahoo.com/group/Forensic Entomology). It is important to note that these groups consisted of forensic entomologist professionals, biologists, medicolegal practitioners, other forensic experts, and people with a general interest in the topic.

**Figure 1.**
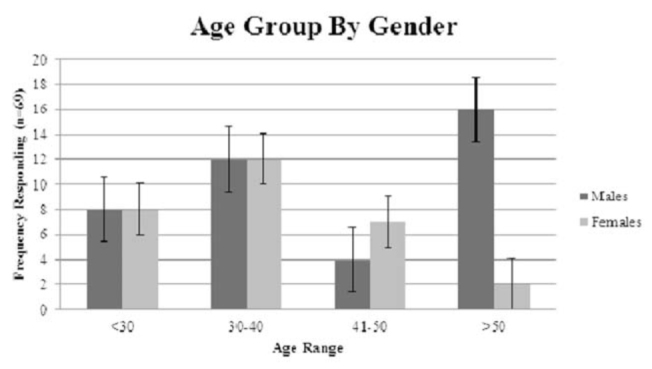
Age group by gender. Analysis showed that the differences across groups was significant (χ^2^ (3) = 10.21, *p* = 0.017). Bars represent standard error. High quality figures are available online.

The questionnaire was conducted between June and September 2009. Responses were analysed by PASW Statistics 18.0 (SPSS, www.spss.com.hk/statistics/) where possible and manually where not. Significance levels for all statistics reported are based on *p* < 0.05, unless exact values are stated.

## Results

Seventy persons responded to the survey (approximately 22% of those contacted). Of those responding to the question of gender (n = 69), 58% were male and 42% were female. While binomial testing showed that this difference was not significant (*z* = 1.33, *p* > 0.05), there were significant differences in gender representations in some of the responses, and those differences are indicated below. Respondents were also asked what age range they were in, and the results are shown in Figure 1.

χ^2^ analysis showed that there was a significant difference in gender representation across age range groupings (χ^2^ (3) = 10.21, *p* = 0.017), with post-hoc binomial testing revealing a significant difference in the representation of 41–50 year-old females (n = 7) compared to males (n = 4) (*p* = 0.27; see Figure 1). Binomial testing also revealed a significant difference between the males (n = 16) and females (n = 2) in the 50-year-old and over category (*p* < 0.001).

### How did they become a forensic entomologist?

Of those responding (n = 69), 43.5% indicated they had obtained a doctorate as their highest level of education, 36.2% indicated they obtained a master's degree as their highest level of education, and 11.6% indicated they had obtained a bachelor's degree as their highest level of education. Two persons (2.9%) indicated they had received no qualification, and 5.8% indicated they had received some other type of qualification. A χ^2^ analysis showed that these qualifications were not significantly different between males and females (χ^2^ (5) = 8.10, *p* = 0.151).

Of those responding (n = 65), 63% indicated that to become a forensic entomologist, their country required some type of qualifications, with 29.2% of those indicating a specialist course was required, 33.8% indicating that a degree was required, and 36.9% indicating that some other qualification was used. In addition to the qualifications required, some countries also had an accreditation process for those in their field (e.g., quality assurance certification (COFRAC 17025) was obtained only in France (Law Enforcement laboratory), Italy (UNI EN ISO 9001:2008) (ASL laboratory, public health service), and Spain (type not defined, university laboratory)), with 69.6% indicating that their country had such processes in place. Of note, there were some discrepancies among respondents from the same countries as to whether their country offered accreditation in the areas of persons, labs, and kits. Those discrepancies are shown in Table 1. Overall, the percentage that indicated their country had a process for the accreditation of forensic entomologists (n = 69) was 30.4%, with the remainder (69.6%) indicating their country had no accreditation scheme. Similarly, the percentage of those that indicated their country had no accreditation processes for their labs (n = 65) or forensic entomology kits (n = 66) was high, at 84.8% and 87.7% respectively. Additionally, 30.4% indicated their government did not provide funding for research in the area of forensic entomology, with 29% indicating their funding for research came from private or other types of funding (n = 69). 40.6% stated their government contributed to research.

**Figure 2.**
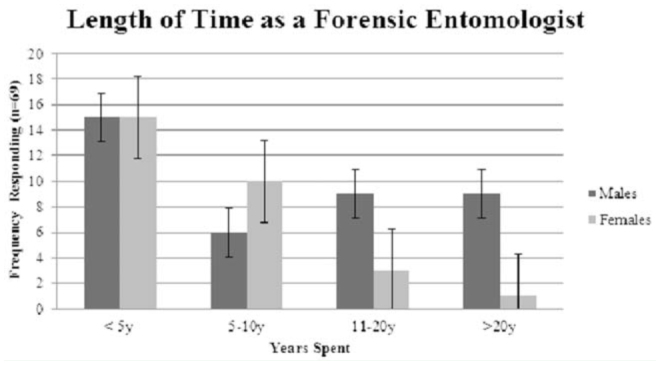
Time (in a range of years) spent as a forensic entomologist, by gender. The difference across groups was significant (χ^2^ (3) = 9.13, *p* = 0.028). Bars represent standard error. High quality figures are available online.

**Table 1.**
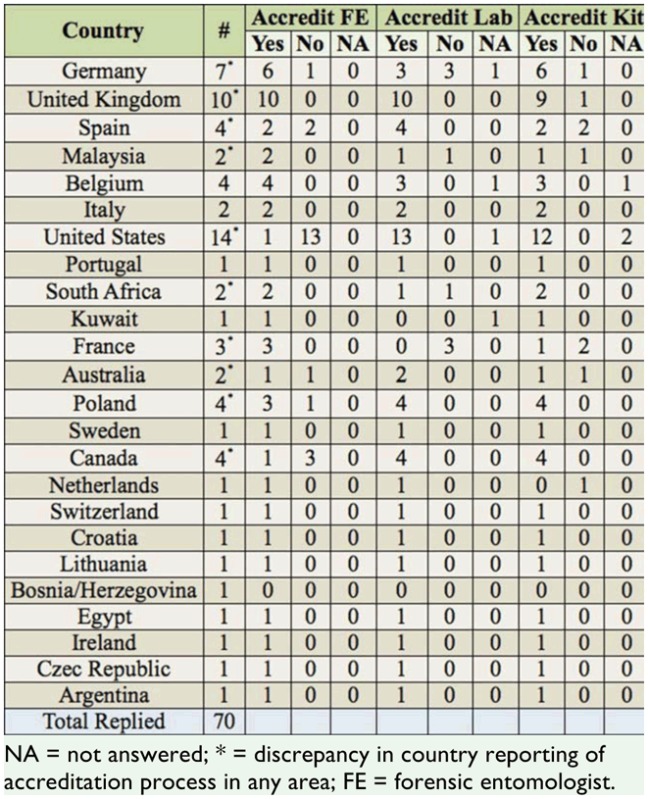
Respondents by country, and their country's accreditation practices in the forensic entomology arena.

**Table 2.**
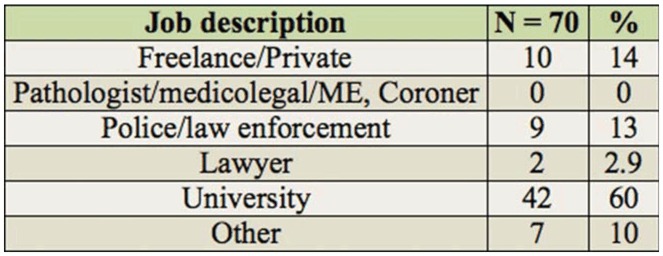
Job descriptions of those responding to survey.

The majority of respondents (n = 69) indicated they were relatively new to the field of forensic entomology, with 44.9% responding they had been a forensic entomologist for less than 5 years. The next largest group, at 23.2%, indicated they had spent 5–10 years in this genre, while 17.4% and 14.5% indicated they had spent 11–21 years and more than 20 years, respectively, as a forensic entomologist, χ^2^ analysis indicated a significant difference between genders across the lengths of time spent as a forensic entomologist (χ^2^ (3) = 9.13, *p* = 0.028), with differences shown in Figure 2.

**Figure 3.**
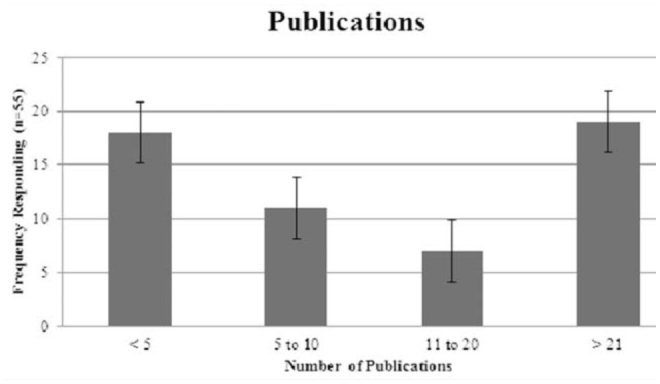
Number of publications for 55 of 70 survey respondents in either a forensic entomology or taxonomy genre. Bars represent standard error. High quality figures are available online.

**Table 3.**
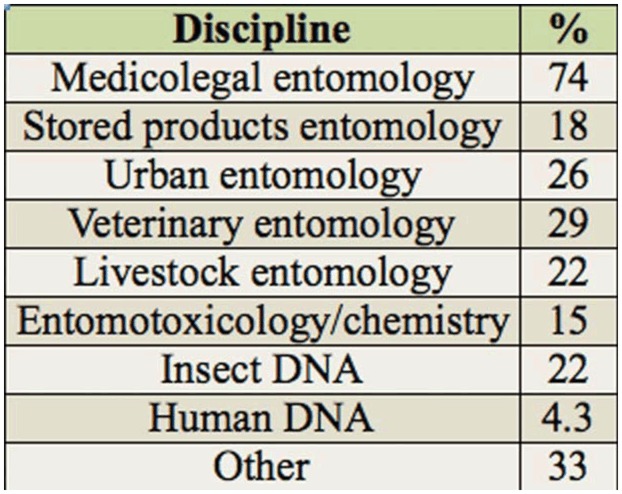
Number of cases worked as a forensic entomologist by number of years in field. Bars represent the number of cases worked. All groups are significantly different (χ^2^ (9) = 46.90, *p* = 0.0001). High quality figures are available online.

### What work does a forensic entomologist do?

Of those responding to the question asking for a description of their occupation (n = 69), the largest proportion indicated that they would describe their occupation as being related to university work (approximately 54%), with the next largest proportions indicating they were privately employed or worked in law enforcement (each with 11.6%). There were equal numbers describing their work as museum related or other types (each with 8.7%), while 5.8% indicated they described their occupation as involving a law enforcement agency. Interestingly, the respondents that indicated they worked as lawyers (see Table 2) did not describe their occupations as such. Furthermore, of those responding (n = 69), 66.7% considered forensic entomology to be their secondary occupation, with the remainder indicating that forensic entomology was considered their first occupation (33.3%). With regards to the scientific discipline the respondents worked in, 42% (n = 69) worked in 1 discipline, with the rest working in 2 or more areas, with 1 person indicating they worked in at least 8 of the 9 areas represented in the survey, and an additional 3 people (4.3%) working in at least 6 areas. The areas and their representations are listed in Table 3.

Additionally, many forensic entomologists indicated they were also active in the publication of their research and other findings, with 78.5% stating they had publication credit in either forensic entomology or taxonomy (see Figure 3).

In terms of workload structure, 47.8% responded that they worked as individuals, with 52.2% responding that they worked as a group member (n = 69), with those groups varying in size from 2 members (21.6%) to large groups (10–20 members, 3.9%) of over 20 members (5.9%), with the largest percentage of respondents working in a group consisting of 3–9 people (68.6%).

### Casework

**Figure 4.**
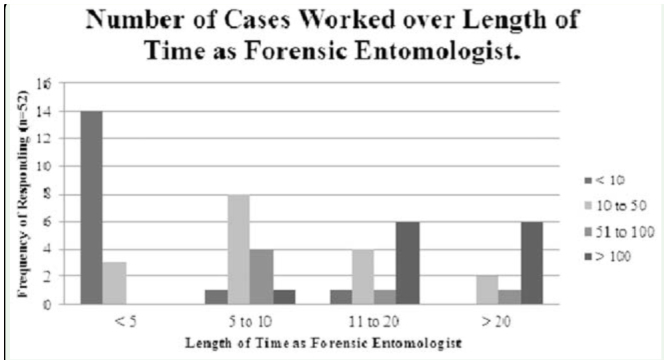
Number of cases worked as a forensic entomologist by number of years in field. Bars represent the number of cases worked. All groups are significantly different (χ^2^ (9) = 46.90, *p*= 0.0001). High quality figures are available online.

To ascertain the procedural aspects of forensic entomology, several questions were asked of and answered by individuals who also conducted casework. Of the 48 who answered, 19.1% of respondents stated they did not attend crime scenes, while 68.8% were invited to crime scenes by the police or other law enforcement agency. Of those who did not attend crime scenes, 44.4% were invited to work on a case by the police or other law enforcement agency, and 22.2% were asked by the pathologist or medical examiner. One person stated they had been asked to work on a case by a prosecuting attorney, and 1 by a defence attorney. Persons responding to the casework section of the survey (n = 52) stated they have worked on a multitude of forensic entomology cases, with 36.5% indicating they have worked on more than 50 cases, and 63.5% having worked on less than 50 cases (see Figure 4), with only 1 person stating they had been asked to conduct casework outside of their country.

Of those responding to conducting casework (n = 55), 67.3% stated they wrote up case reports, 18.2% did not, and 14.5% indicated they sometimes did. When asked if there was a standardised format for those case reports, 45.8% (n = 48) indicated they did not use a standardised format, while 54.2% did. Binomial testing indicated that this difference was not significant (z = 0.07, *p* > 0.05). When asked if required to give evidence, 53.7% indicated they did not (n = 54), despite 79.2% of respondents stating their country required/invited them to attend homicides (n = 48). 82.4% (n = 34) were required to attend suspicious deaths, and 81.3% (n = 54) were required to attend cases of neglect.

## Discussion

As indicated in the introduction of this paper, there are huge inequalities in the forensic sciences, including forensic entomology, and the shortcomings highlighted in the practices of the forensic sciences in the United States are echoed in the findings in this study. The results of the survey indicated there were also vast disparities across many areas surveyed.

One area of great concern is the education obtained by practicing forensic entomologists. While it may seem encouraging that nearly 80% of those responding had some type of formal postgraduate education (e.g., Masters or Doctorate), it is noteworthy that the remaining proportion had the equivalent of a bachelor's degree or less. This disparity in education is problematic for several reasons. First, people with postgraduate degrees, especially PhDs, would have a more holistic perspective of the science. Second, the lack of scientific training at undergraduate and technical levels may thwart any interpretation of the data within the literature and hence application will be substandard. Third, a lack of education hinders research, an important aspect of the advancement of the field of forensic sciences, and especially the advancement of the entomologist domain. Amendt et al. (2004) stated that one of the more important considerations in the advancement of the science is the integration of experimental and practical casework, and research is an integral component of that. In addition to the observations of Amendt et al. (2004) is the NAS (2009) finding that research in the forensic sciences was severely underfunded, leaving many disciplines grappling with the burdens of advancing their science on a shoestring budget. Many postgraduate programs incorporate a healthy dose of research based components, and persons lacking in this vital area of education may lack the skills for the proper handling and interpretation of such data, or even fail to understand the importance of increasing the number of detailed and quantifiable research and publications. Fortunately, of those responding to this survey with regards to publications, over two-thirds indicated that they had publication credits in the fields of forensic entomology or taxonomy.

Over half of responders indicated that they had been asked to give expert testimony at times, with a large majority being asked to attend homicides or other events with legal ramifications (i.e., neglect, etc.) by police or other law enforcement agencies. This highlights the significance of the forensic entomologist in the procurement and interpretation of evidence and underscores the necessity of an accreditation process.

This survey emphasized a lack of congruency in the accreditation and certification processes globally, and more importantly within each country. That is, over half of the respondents indicated that their country required some type of accreditation for personnel, facilities, and collection kits. Alarmingly, some respondents within the same country denied knowledge of accreditation or certification processes required to practice forensic entomology. The obvious and immediacy with which this issue in particular must be rectified should not be overlooked. The very credibility of forensic entomology as a science relies on the communication and consistency of the certification of persons, facilities such as laboratories, and importantly, evidentiary collection kits, being communicated within the country of jurisdiction. Failing at this, the process by which the scientific-method that is expected by judiciary standards, such as the Daubert Criteria, becomes ineffectual.

Additionally, the forensically important area of casework was assessed. This too had varying practices across the survey responders. Over two-thirds responded that they wrote up case reports, while the remainder indicated that they either occasionally wrote reports or did not write reports at all. This result suggests that the vital component of casework reporting also lacks continuity across the practice, and indeed even across the individual's local practice concerning casework. In addition to the reporting of the casework, there were no statistical differences between those that utilized a standard format for their casework reporting, as the results were about half and half. Interestingly, no country with multiple respondents indicated they utilised a standardised format throughout, indicating that those forensic entomologist that did use a standardised format for their casework reporting may have done so as a local strategy (i.e., personal or departmental policies). This was further highlighted at the recent European Association of Forensic Entomology conference in Poland, which endorsed the policy that information gained from a global standardized report should be used for a future accreditation process (Amendt et al. 2012).

The gap between 1986, the publication of the first survey, and the 23 subsequent years until the second survey was probably too long. The recent reports in the US concerning experts and their requirements in core disciplines such as DNA and fingerprint evidence have emphasized that we can no longer quietly present entomological evidence solely based on accumulated merits. Moreover, the Boards and Societies within this discipline need to think about mechanisms and requirements to accredit the expert testimony of forensic entomologists. The authors would hope that this information will help change current attitudes, and that at the end of this decade we can reflect back on the status of the discipline with another, more intensive questionnaire.
